# Dental Chemistry of the Mouth

**Published:** 1856-12

**Authors:** Geo. Watt


### DENTAL CHEMISTRY OF THE MOUTH.

BY GEO. WATT.

As the morbid condition which the dental surgeon is most frequently called upon to treat, is now universally conceded to be the result of chemical action; and as the most important point to be noticed, in replacing the parts lost by its devastations, is the selection of such substances as will neither act chemically on any of the substances within the mouth, nor be acted upon by them; it follows, that the chemistry of the mouth in all its bearings, invites our closest study and the most careful scrutiny. We propose, therefore, to devote some attention to the subject, without any attempt at originality or depth of thought. If we can present the subject in such light that a majority of our readers can comprehend our meaning; and, especially, if we can render our remarks practically useful, our highest ambition will be gained. And, we may as well state here, that we do not intend to be either very systematic or technical. We know that many of our readers are not practical chemists; and, while they think and reason so clearly for us on other subjects, it will be but small recompense if we can be useful to them in this.

In the discussion of the subject, we will avail ourselves of the light of experiments, and of the thoughts of others.

The teeth are composed of three distinct anatomical elements; the *enamel*, the *cementum*, or *crusta petrosâ*, and the *dentine.* These differ from each other, both in physical structure and chemical composition. Each is composed of animal matter and earthy salts. The dentine constitutes the principal bulk of the tooth, and, will therefore, receive our first attention.

The proportions of organic and inorganic matter vary with the constitution, temperament, and age. As life advances, the calcareous matter is increased. Hence, any two or more analyses, are not likely to agree in all particulars.

[illegible]erzelius found in human dentine:

| | |
|---|---|
| Cartilage and Vessels, | 28.0 |
| Phosphate of Lime, with Fluoride of Calcium, | 64.3 |
| Carbonate of Lime, | 5.3 |
| Phosphate of Magnesia, | 1.0 |
| Soda, with Chloride of Sodium, | 1.1 |
| Loss, | .3 |
| | 100.0 |

It is needless to multiply analyses, as this is, doubtless, sufficiently reliable for practical purposes. But, that we may the better understand the subject, it may be profitable to consider briefly, the chemical characteristics of each of the above ingredients.

PHOSPHATE OF LIME, as its name imports, is composed of phosphoric acid and lime. As the acid and the base unite in several proportions, more than one phosphate is formed by their union. As these differ in important respects, it is important to know which of them is a constituent of tooth-bone.

Phosphoric acid is formed by the union of about 32 parts of phosphorus, with 40 of oxygen. Its formula, is $P_2 O_5$. Lime is composed of 20 parts of the metal, calcium, and 8 of oxygen, and its formula is CaO. Now, the phosphate found in bony tissues, is composed of 224 parts of lime, united with 214 of the acid; and its formula is $8CaO + 3 P_2 O_5$. This is not the neutral phosphate, but is often called the subphosphate, and, still more frequently perhaps, the "bone phosphate." It may be obtained pure by calcining the teeth (or bones), in a crucible, to complete whiteness, reducing to powder, and digesting with hydro-chloric acid. The bone phosphate is dissolved in the acid, and may be precipitated from it by caustic ammonia.

The sub-phosphate thus obtained, is a white, tasteless, inodorous powder, insoluble, in water ; but soluble in acetic, nitric, and hydro-chloric acids. It may be thrown down, from any of these solutions, unchanged in composition, by ammonia, potash, and their carbonates. It is a very permanent compound being fused, but not decomposed by the most intense heat.

CARBONATE OF LIME, is composed of carbonic acid and lime. Carbonic acid is so well known, that it need not be described. Its symbol is, $CO_2$ ; and, hence the formula of this carbonate is CaO, $CO^2$. It exists abundantly in the forms of chalk, marble, &c., and is very readily decomposed by most acids, and by heat.

PHOSPHATE OF MAGNESIA, is composed of phosphoric acid and magnesia. Its quantity in the tooth is not sufficient to modify practically, any chemical action liable to take place in the mouth.

SODA, is the well known alkali, composed of 8 parts of oxygen, combined with 23 of the metal sodium. It possesses a a strong affinity for acids; but its quantity is so limited that it need scarcely be mentioned in considering the chemical actions by which the tooth is destroyed.

CHLORIDE OF SODIUM, is the common table salt; and, though it sometimes plays an important part in the chemistry of the mouth, yet but a trace of it is found in dentine, and hence, we will omit its consideration for the present.

The *cementum* or *crusta petrosa*, contains more organic matter in proportion to its inorganic, than dentine. Its composition, according to Lassaigne, is :—

| | |
|---|---|
| Organic Matter, | 42.18 |
| Phosphate of Lime, | 53.84 |
| Carbonate of Lime, | 3.98 |
| | 100.00 |

As the cementum has less density than dentine, it is more readily acted on by chemical agents; but while the mouth retains its normal structure, it is not exposed to either the ordinary or incidental chemical influences within the mouth. Its constituent substances were explained in speaking of dentine.

The enamel, according to Berzelius, is composed of :

| | |
|---|---|
| Phosphate of Lime, with Fluoride of Calcium, | 88.5 |
| Carbonate of Lime, | 8,0 |
| Phosphate of Magnesia, | 1.5 |
| Membrane, alkali, and Water, | 2.0 |
| | 100.00 |

The extreme hardness of enamel protects it from the action of chemical agents, which would act readily on substances of the same composition, with less density.

The animal portion of the tooth is possessed, in general, of the same chemical composition, as other organic parts of the body. Albumen, fibrin, and casein, constitute the principal bulk of animal bodies. It is now generally conceded, that these are but modifications of one and the same compound, which is denominated *protein*, and which is regarded as the starting point of all the tissues. Its composition, according to Mulder, and others, is represented by the formula, $C_{40}\ H_{31}\ N_5\ O_{12}$. Its equivalent on the hydrogen scale, is 442.4.

The organic tissue of bone, cartilage, &c., is generally included under the term *gelatin;* but strictly speaking, this substance does not exist in the animal tissues, but is formed from them by the action of boiling water.

The organic portion of the tooth is liable to decomposition from various sources. If its vitality be lost, of course it undergoes spontaneous decomposition, unless prevented by antiseptic measures. The alkalies form with it soluble compounds; and most of the acids, if concentrated, or in their nascent state, are capable of decomposing it, by virtue of their affinities for some of its constituents.

Water, of course, is an ingredient of the vital portion of the tooth, as of all the other organic tissues. In some of the chemical phenomena which occur in the mouth it plays an important part. Every one knows that it is composed of oxygen and hydrogen,—eight of the former to one of the latter.

Having now briefly noticed the chemical structure of the teeth themselves, the next thing is the chemical characteristics of the various agents, either ordinarily or incidentally brought in contact with them, which are, or may become capable of acting injuriously on them. The first that we will consider is the saliva.

SALIVA.—The saliva has, of late years, received much attention from chemists, yet the subject is still somewhat complicated and obscure. It is not our purpose to give a full, or even a lengthy notice of it. "According to Dr. Wright, pure saliva is a limpid fluid having a faint blue tinge, and a slight degree of viscidity. It is perfectly uniform in consistence, and unobscured by frothiness or floculi."

The saliva contains but a very small quantity of solid matter. This is composed of spirit and water extracts, ptyalin, fat, albumen, sulphocyanogen, and a variety of salts. These salts are usually phosphates, carbonates, lactates, traces of sulphates, and chlorides.

"Berzelius found in 1000 parts of human saliva:

| | |
|---|---|
| Water | 992.9 |
| Ptyalin | 2.9 |
| Mucus | 1.4 |
| Extract of flesh, with alkaline lactates | .9 |
| Chloride of Sodium | 1.7 |
| Soda | .2.' |

Simon found in 1000 parts of his own saliva:

| | |
|---|---|
| Water | 991.22 |
| Solid constituents | 8.77 |

These solid constituents were:

| | |
|---|---|
| Fat containing cholesterin | .52 |
| Ptyalin, with extractive matter | 4.37 |
| Extractive matter and salts | 2.45 |
| Albumen, mucus and cells | 1.40 |

The saliva varies very considerably in its composition, and, no doubt, many changes may take place without a forfeiture of its right to the title of *normal* saliva. Then, its abnormal states are represented by a great variety of modifications.

The chemical agents in healthy saliva most important to be noticed, at least as far as the *dental* chemistry of the mouth is concerned, are water, the chlorides, sulphocyanogen and soda.

Any one who will take the time and pains can, by observing a few plain directions, test the saliva, in any given case, with sufficient accuracy for practical purposes. And the first thought that strikes the operator is likely to be, "Is the saliva acid, alkaline, or neutral?" This can be usually determined with sufficient accuracy by testing with prepared litmus paper. And every practitioner should have a supply of "test papers," in his case, ready for use on any occasion that may require them. Nor should he rest satisfied with merely a general examination; but if, for example, he discovers acidity in the mouth he should go on to ascertain its source, whether it be in the mucus, the saliva, or in decomposing foreign substances. If it be in the saliva, he should ascertain whether it be in the secretions of all the glands, or confined to but one or more of them. All this he must know, and much more, before he can be at all prepared to treat intelligently any case in which the secretions of the mouth are abnormal.

The saliva may be tested for albumen, by the application of heat. The albumen, if present, will be coagulated and render the liquid turbid. Sulphocyanogen is indicated by the redness produced by the addition of perchloride of iron. Simon says: "With a view to separate the constituents of the saliva, I evaporated a known quantity to dryness, and thus determined the water. I then treated the residue with ether, for the purpose of extracting the fat; and with water, in order to take up the ptyalin, extractive matter and salts. The insoluble residue that had resisted the action of ether and water, consisted of albumen and mucus. Another portion of the saliva was decanted from its precipitate, evaporated to a small residue, and the ptyalin with a trace of extractive matter, precipitated by alcohol."

The specific gravity of healthy saliva varies considerably. It is generally, if not always, a little above that of water.

Pure saliva absorbs oxygen, which contributes to its digestive power. Dr. Wright found that saliva, which had been exposed some hours to an atmosphere of oxygen, converted a much greater quantity of starch into gum and sugar, than saliva not so exposed.

MORBID SALIVA.—The saliva is liable to a variety of abnormal modifications. It is not our purpose, at present, to notice all of these conditions, nor any of them at any great length.

As before intimated, a free acid, or more than one, may be found in the saliva. The lactic is the most common, but acetic, hydrochloric, oxalic, uric, and others are sometimes found. The acid reaction is easily detected by test paper. Normal saliva, being alkaline, imparts a blue tint to red litmus paper, while this reddens blue paper. If, on evaporation to dryness, the acid reaction is destroyed, it is evident that the free acid is not the *lactic*, which is the one most frequently found. Hydrochloric acid may, generally be detected by its action on a fresh solution of nitrate of silver; but as the soluble chlorides, if present in the saliva, would produce similar results, the acid may be separated from them, and from most of the other constituents of the saliva, by distillation. Its quantity may then be estimated by the amount of chloride of silver which constitutes the precipitate. Chloride of silver is represented by the formula Ag. Cl. and is composed of 108 parts of silver to 35 of chlorine. The 35 parts of chlorine required 1 part of hydrogen, to form hydrochloric acid; hence, when the weight of the chloride is ascertained, the calculation is easy.

According to Dr. Wright, "The saliva is impregnated with *lactic acid*, chiefly in gout, rheumatism, ague, diabetes, and gastro-enteritis; with *acetic acid* in apthae, scrofula, scorbutus, small-pox, protracted indigestion, and after the use of ascescent wines; with *hydrochloric acid* in simple gastric derangement from immoderate or improper animal food; and with *uric acid* in gouty affections." He tells us also that acidity of the saliva is likely to occur in fevers, both typhoid and inflammatory; in measles, phthisis, venereal disease, many skin diseases, catarrhs, mumps, and in the tedious dentition of weakly children.

After noticing the chemical properties of the mucus of the mouth, and the characteristics of some other agents frequently in contact with the teeth, we will endeavor, in a future number, to set forth the various relations which these several agents sustain to each other, and make a practical application of the various facts and principles referred to.

